# Coffee Drinking and Adverse Physical Outcomes in the Aging Adult Population: A Systematic Review

**DOI:** 10.3390/metabo12070654

**Published:** 2022-07-15

**Authors:** Simon Mazeaud, Fabio Castellana, Hélio José Coelho-Junior, Francesco Panza, Mariangela Rondanelli, Federico Fassio, Giovanni De Pergola, Roberta Zupo, Rodolfo Sardone

**Affiliations:** 1 UFR of Biology, Campus Universitaire des Cézeaux, University of Clermont Auvergne (UCA), 63000 Clermont-Ferrand, France; simon.mazeaud@sfr.fr; 2 Unit of Data Sciences and Technology Innovation for Population Health, Department of Basic Medicine, National Institute of Gastroenterology “Saverio de Bellis”, Research Hospital, 70013 Castellana Grotte, Italy; fabio.castellana@irccsdebellis.it (F.C.); rodolfo.sardone@irccsdebellis.it (R.S.); 3 Applied Kinesiology Laboratory-LCA, School of Physical Education, University of Campinas, Campinas 13083-970, Brazil; coelhojunior@hotmail.com.br; 4 Department of Geriatrics, Neurosciences, and Orthopedics, Teaching Hospital “Agostino Gemelli”, Catholic University of the Sacred Heart, 00168 Rome, Italy; 5 Neurodegenerative Disease Unit, Department of Basic Medicine, Neuroscience, and Sense Organs, University of Bari Aldo Moro, 70013 Bari, Italy; f_panza@hotmail.com; 6 Unit of Human and Clinical Nutrition, Department of Public Health, Experimental and Forensic Medicine, University of Pavia, 27100 Pavia, Italy; mariangela.rondanelli@unipv.it; 7 Unit of Biostatistics and Clinical Epidemiology, Department of Public Health, Experimental and Forensic Medicine, University of Pavia, 27100 Pavia, Italy; federico.fassio01@universitadipavia.it; 8 Unit of Geriatrics and Internal Medicine, National Institute of Gastroenterology “Saverio de Bellis”, Research Hospital, 70013 Castellana Grotte, Italy; giovanni.depergola@irccsdebellis.it

**Keywords:** coffee, physical functioning, frailty, sarcopenia, gait, mobility, aging, adult population

## Abstract

Declining physical functioning covers a prominent span of later life and, as a modifiable driver to be leveraged, lifestyle plays a critical role. This research aimed to undertake a systematic review investigating the association between levels of coffee consumption and declining conditions of physical functioning during aging, such as sarcopenia, frailty, weakness, falls, and disability, while trying to explain the underlying mechanisms, both from a metabolic and social angle. The literature was reviewed from inception to May 2022 using different electronic databases, not excluding the grey literature. Two independent researchers assessed the eligibility of 28 retrieved articles based on inclusion criteria; only 10 met the eligibility requirements. Different levels of coffee consumption were considered as exposure(s) and comparator(s) according to PECO concepts, while middle age was an inclusion criterion (40+ years). No limitations were set on the tool(s) assessing physical functioning, type of dietary assessment(s), study setting, general health status, country, and observational study design (cohort, cross-sectional). The cross-sectional design outnumbered the longitudinal (90%, n = 9/10). The overall quality rating was judged poor (70%) to good (30%). It was found that higher exposure to coffee drinking is strongly associated with better physical functioning outcomes, and the findings showed consistency in the direction of association across selected reports. Countering physical decline is a considerable challenge in easing the burden of population aging. For preventive models that aim to allow a better lifestyle, it has to be kept in mind that increased coffee consumption does not lead to poor physical functioning.

## 1. Introduction

Population aging is a major challenge and top priority in the 21st century. As the number of older people in industrialized countries grows, the World Health Organization (WHO) underlines how important it is for aging adults to keep their physical mobility so that they can continue to live active, independent lives [1]. Indeed, this population subset is far more likely to suffer functional impairment [2,3], frailty [4], and disability [5,6]. A systematic review report of studies across several countries by Collar and colleagues [7] found a 10% and 25% prevalence of frailty in community-dwelling people aged over 60 and 80 years, respectively. We recently found comparable prevalence data in our “Salus” study of an elderly population in southern Italy [3]. In the United States, 50% of people aged 80 and older reported mobility limitations, 35% some disability in instrumental activities of daily living (IADL), and 27% some disability in basic activities of daily living (ADL) [8]. Preventing the adverse physical outcomes induced by the progressive age-related deterioration of the musculoskeletal system is critical to increasing the number of healthy life years, avoiding institutionalization, and reducing the healthcare system burden imposed by the geriatric population subset. To this end, a better understanding of lifestyle modifiable risk drivers of sarcopenia, frailty, loss of mobility and autonomy, falls, weakness, disability, and muscle vigor loss appears critical to improving monitoring and prevention in older people.

Epidemiological research into the association between dietary factors and bad physical outcomes in the aging population is poor, still lacking in evidence, and mainly focuses on antioxidants, B vitamins, fruits and vegetables, and dietary patterns [9,10,11,12,13]. Beverages fall into the class of dietary consumption essentials, which is a hot topic, especially in aging populations that undergo physiological alterations in thirst and taste. With an estimated 2.25 billion cups drunk daily worldwide, coffee is one of the most widely consumed beverages in the world [14,15]. Population studies suggest that coffee consumption is highly prevalent among the elderly [16]. Coffee drinking provides exposure to a huge number of biologically active compounds and nutrients [17], such as polyphenols, lipids, minerals, and particularly caffeine, which is the most widely consumed psychoactive substance in the world (85% of the US population) [18]. Improvements in a wide variety of health outcomes due to exposure to coffee consumption have already been described in the literature, including lower mortality, weight, cancer, diabetes, or patterns of markers of inflammation and insulin resistance [14,19,20,21,22], often showing a dose-dependent relationship [23]. For all these reasons, coffee consumption has attracted and continues to attract an enormous amount of research.

Against this background, assessing the association between exposure to coffee consumption and the physical decline outcomes of aging seems to be an important issue. Unfortunately, to our best knowledge, the current literature lacks an overview of this research question. The present research aimed to assess the magnitude and direction of the association between different coffee exposure levels and risks of adverse physical outcomes, including physical frailty, sarcopenia, impaired walking and mobility, and disability. Findings would help summarize the available window of evidence to improve public health advice on coffee consumption in the aging adult population while trying to unfold possible underlying mechanisms, both from a metabolic and social perspective.

## 2. Results

**Figure 1:**
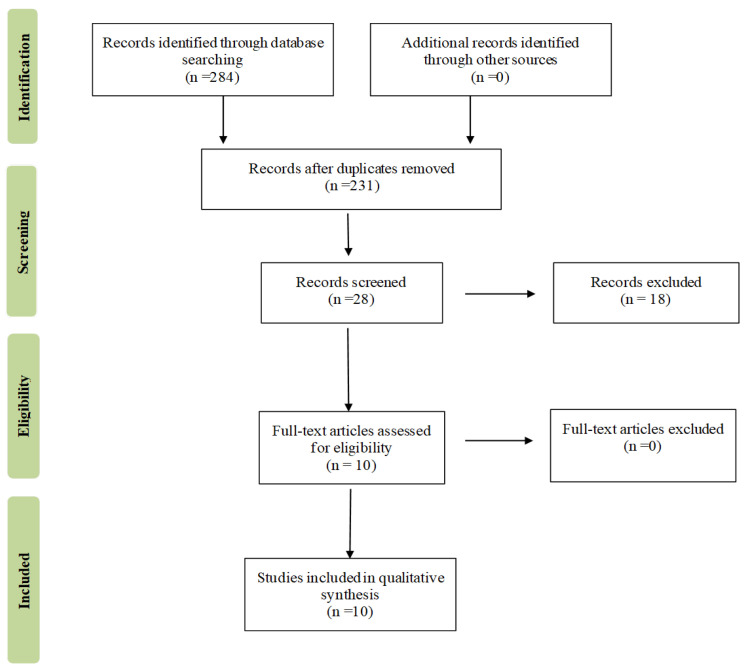
Preferred reporting items for systematic reviews and meta-analyses (PRISMA) flow chart illustrating the number of studies at each stage of the review.

**Figure 2:**
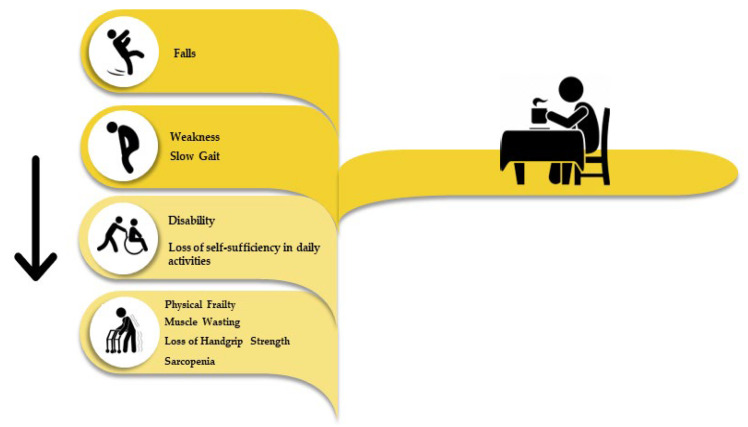
Graph overview of the results.

The first systematic search of the literature yielded 284 entries. After excluding duplicates, 231 were classified as potentially relevant and selected for the title and abstract analysis. Then, 203 were excluded for failing to meet the characteristics of the approach or the review goal. After reviewing the full text of the remaining 28 records, only 10 met the inclusion criterion of age and were included in the final qualitative analysis [24,25,26,27,28,29,30,31,32,33]. The flow chart of Preferred Reporting Items for Systematic Reviews and Meta-analyses (PRISMA), illustrating the number of studies in each review stage, is shown in Figure 1. The final study base included ten articles reporting nineteen different outcomes. Figure 2 shows a graph overview of the results.

Details of the design (cohort, cross-sectional), sample size (N) and sex ratio (%), minimum or age range, study population, and the country of each study are provided in Table 1. The cross-sectional (90%, N = 9 out of 10) predominated over the longitudinal design [30]. The recruitment contexts were community-based, except for one that collected data from universities, colleges, and technical schools [27] and one in a hospital context [33]. The geographical setting of the selected studies was evenly distributed between Asia (N = 5/10, 50%) and Europe (N = 4/10, 40%), with an American minority [24].

Following the inclusion criteria, subjects were over 40 years of age, predominantly 60 or older. Of the 34,921 individuals in the selected studies, the female sex was more prevalent, yet many studies failed to report the sex ratio. Regarding those outcomes fitting the inclusion criteria, the selected studies (N = 10) reported a set of adverse physical conditions, i.e., poor daily living skills (i.e., lower extremity mobility, general physical activity, leisure and social activities, impaired agility, impaired mobility, and impaired general physical function), sarcopenia (according to operational construct(s) or individual dimension), frailty (according to Fried’s phenotype or the FRAIL scale), falls, slow gait (assessed by global gait or gait speed), and exhaustion (assessed by SPPB or chair rise test). The main finding was the consistent direction of the association, in the sense that the greater the coffee consumption, the better the physical functioning (Table 2).

Concerning outcomes of poor daily living skills, we found articles by Wang [24], Machado-Fragua, and colleagues [30] reporting on the association between coffee consumption and lower extremity mobility, general physical activity, leisure, and social activities, and declines in activities of daily living or instrumental activities of daily living. The findings were roughly consistent in that direction. On the one hand, authors found that higher coffee consumption reduced odds of functional disability in older U.S. adults; on the other hand, no association suggested an increased risk of functional disability. Indeed, higher coffee consumption could even benefit women and patients with hypertension, obesity, or diabetes.

Regarding sarcopenia, three studies reported on this outcome, but only one focused on individual dimensions of sarcopenia. Chung and Kim’s reports [25,26] collected data on the KNANES Korean population, exploring ASMI as a measure of sarcopenia. They found that the consumption of at least 3 cups of coffee daily was associated with a lower prevalence of sarcopenia in older Korean men. On individual dimensions of sarcopenia, i.e., SMI and handgrip strength, Iwasaka and colleagues [28] found a significant positive correlation between coffee intake and SMI levels. In contrast, handgrip strength did not reach statistical significance, although a positive trend was reported. Similarly, Jyvakorpi and colleagues [33] found a linear, non-significant association between coffee consumption and handgrip strength.

A single report evaluated the risk of falls as an outcome [32], reporting that habitual coffee consumption was associated with a lower risk of falls in two European cohorts, i.e., older people in the Seniors-ENRICA cohort (Spain) and the UK Biobank study. Last, Verlinden [31] and Jyvakorpi [33] reported on gait speed and exhaustion. They documented associations between coffee consumption levels and overall gait, gait speed, SPPB, and chair sitting and standing test, respectively. Verlinden concluded that in a community-dwelling population, consuming more than one daily cup of coffee is related to a better gait; consistently, Jyvakorpi found the same positive trend with gait speed and handgrip strength, SPPB score, and sitting and standing test points.

**Figure 3:**
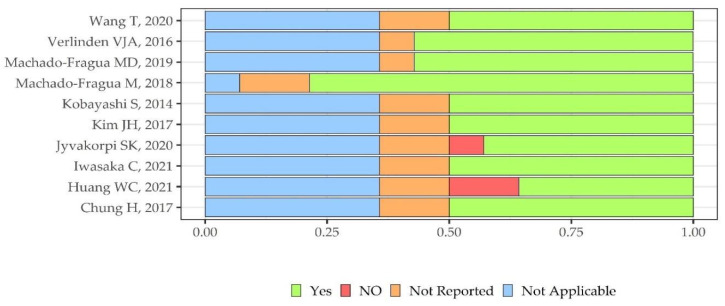
Quality assessment plot across selected studies N= 10.

We found a poor (N = 7), fair (N = 2) to good (N = 1) methodological quality overall. An overview of quality ratings within and across studies is provided in Supplementary Table S2 and Figure 3, respectively, highlighting areas with higher or lower ratings. Biases were found mainly in the domains of sample size justification (selection bias) and blinded assessors (detection bias) (91% and 82% of studies, respectively), and to a lesser extent in the domains of different levels of exposure (46% of studies) and multiple exposure assessments over time (73% percent of studies) in light of the prevalent cross-sectional setting. Since 73% of the studies had a cross-sectional design, the same percentage reflected an unclear risk for the following qualitative assessment items: prior exposure to the outcome, sufficient time frame, and loss to follow-up.

## 3. Discussion

The present systematic review addressed the conceptual hypothesis of a link between coffee consumption and better outcomes in terms of declining physical functioning in the aging adult population. To this end, the body of evidence on different exposure levels to coffee consumption was examined against a cluster of impaired physical functioning outcomes, as assessed by operationalized constructs and other validated tools related to sarcopenia, frailty, exhaustion, gait, falls, and disability. The most important finding was the consistent direction of association across all studies selected to fill the knowledge gap about the research question. Although most reports had a cross-sectional design, thus leaving little room for causal inference, we found that the higher the coffee consumption, the greater the drop in adverse outcomes of physical functioning. The above negative link between coffee consumption and adverse outcomes of physical functioning may be explained from a social perspective as well as, conceivably, from a causal, biological, and metabolic standpoint in the context of aging.

Physiologically speaking, aging occurs with a pattern of sensory decline [3,34], translating into reduced appetite and sensory perception, which are well-known dimensions underlying frailty, sarcopenia, and physical decline [35,36]. This sensory deficiency carries serious implications for safety, nutrition, quality of life, and social relationships [37]. On this latter point, impairing physical functioning easily leads to a cluster of social deprivations with a concurrent steady loss of conviviality and social drinking opportunities. In other words, the less physically fit you are as you age, the less likely you are to drink “social” coffee or other drinks in company with other people.

From a biological perspective, previous evidence consistent with our findings points to the protective bromatological properties of coffee that promote physical well-being. Both animal and human reports discuss putative mechanistic explanations of a causal protective effect of coffee on physical and musculoskeletal health in aging. Guo and colleagues found, in aged mice, a preventive in vivo effect of coffee treatment on sarcopenia progression, along with an increase in muscle mass, grip strength, and regenerating capacity of injured skeletal muscles, likely explained by a decrease in low-grade systemic inflammation, which is one of the causative drivers of sarcopenia, thanks to the antioxidant and anti-inflammatory properties of coffee drinking [38]. The same report found increased proliferation rates, DNA synthesis, and activation of the Akt signaling pathway in satellite muscle cells of coffee drinkers [38]. Jang and colleagues found the same muscle hypertrophy in mice, possibly explained by a decrease in transforming growth factor-β (TGF-β) myostatin while increasing insulin-like growth factor (IGF) expression [39]. Furthermore, coffee bioactive has been shown to improve insulin sensitivity and muscle glucose uptake [40].

On the other hand, the loss of muscle mass and functionality in the aging population can be partially attributed to a loss of digestive functions, enzyme production [41], and appetite, thus causing malnutrition [42], especially regarding the availability of amino acids for protein synthesis. Coffee is known to stimulate digestive activity [43]. Recent randomized control trials (RCT) indicated a significantly higher salivary alpha-amylase production due to coffee drinking. It stimulates gastric secretions, gallbladder secretions, and pancreas secretions. These findings could be, in the majority, associated with caffeine effects.

The best mechanistic explanation is an indirect effect of coffee on physical health. Physical activity is one of the most effective ways to maintain health and prevent physical decline, so feeling tired and lacking energy may be a hindrance. Caffeine is a well-established strong ergogenic aid whose performance-enhancing effects on strength and endurance have been documented in a wide range of physical tasks. Torquati and colleagues found that consuming 1–2 cups of coffee per day is associated with a 17% increase in the likelihood of meeting physical activity guidelines in middle-aged women, undoubtedly due to the caffeine increasing energy levels and reducing fatigue.

Despite the fact that further trials are needed to corroborate the causal path, caffeine and some of its metabolites, including the main one, paraxanthine, show notable potential pharmacological interests, sometimes different from caffeine, for example on nitric oxide (NO) neurotransmission [44,45]. Jäger and colleagues showed interesting results of paraxanthine supplementation on grip strength, muscle mass, treadmill performance and NO in mice, all with seemingly less toxicity compared to caffeine [45,46]. On the other hand, theophylline has already shown an effect of reducing susceptibility to fatigue by improving ventilatory functions through its bronchodilator effect and on the contractility of the diaphragm [47] and is commonly used in the elderly in the context of asthma or chronic airway diseases [48]. Like caffeine, its effect has also been shown on physical performance. Despite an heterogenous literature [49,50] and discussed toxicity [46], extending theophylline, but also paraxanthine research into the prevention and treatment of negative physical outcomes, is of interest because of its prevention of exhaustion and its ergogenic effect.

Lastly, in a public health perspective, and based on the coffee’s beneficial effects so far reported for non-communicable degenerative illnesses of aging such as cancer, cardiovascular disorders, diabetes, Parkinson’s disease, and cognitive impairment [41,42], consuming 2 to 3 cups of coffee may be protective against chronic disease occurrence, and therefore the functional deterioration closely linked to multimorbidity and polypharmacy in aging population settings.

We acknowledge some limitations of this systematic review that could create critical bias. Firstly, coffee and the preparation methods are not the same worldwide. It has already been noted that preparation methods could impact cup content and coffee effects. For instance, filtered coffee is free of the diterpenes cafestol and kahweol, which are present in non-filtered coffee and could impact carcinogenic and cholesterolemic effects [43]. Unfiltered coffee is the coffee most commonly consumed in Spain, whereas, in the United States, coffee is consumed mainly after filtering. Only one of the selected ten studies performed a different analysis between caffeinated and decaffeinated coffee, thus discriminating the effect of caffeine’s primary bioactive component. Furthermore, the selected studies did not stratify their analyses to include coffee consumption above 3 cups or 330 g per/day, and many stopped at 2 cups per day while a dose–response relationship is well-acknowledged [23]; thus, the opposite results with higher consumption are possible. Strengths include the cluster of physical functioning outcomes, embodying extensive evidence on the topic. Moreover, accounting for different levels of exposure to coffee consumption as comparators adds value to these research findings.

## 4. Methods

### 4.1. Search Strategy and Data Extraction

This systematic review adhered to the Preferred Reporting Items for Systematic Reviews and Meta-Analyses (PRISMA) checklist of 27 items [51]. An a priori protocol for the search method and inclusion criteria was conceived and registered on PROSPERO, a prospective worldwide register of systematic reviews, with no modifications to the information supplied at registration (CRD42023338863). Ethical review and approval were waived for this study because a revision is involved and not an original article.

We conducted separate searches in the US National Library of Medicine (PubMed), Medical Literature Analysis and Retrieval Online (MEDLINE), EMBASE, Scopus, Ovid, and Google Scholar databases to identify original articles investigating any possible association between coffee exposure and adverse physical outcome(s). The primary goal was to determine whether different exposures (comparators) to coffee consumption, as measured by dietary intake (cups per day, rising quintiles of daily consumption, or grams per day), were associated with unfavorable physical outcomes in the aging adult population. We also reviewed the gray literature at the study selection stage. To pinpoint abstracts of significant conferences and other information that specialists had not evaluated, we turned to the largest preprint repository, https://arxiv.org/ (accessed on 12 May 2022), as well as the database http://www.opengrey.eu/ (accessed 12 May 2022). We also aimed, particularly in the grey search step, https://www.base-search.net/ (accessed on 12 May 2022) to eliminate publication bias in contradictory and unfavorable results. Since we chose to include only observational studies, the search strategy followed the PECO (Populations, Exposure, Comparator, and Outcomes) concepts [52]. Thus, we took into account populations (only adults, 40 years or older population), exposure (coffee intake or consumption), comparators (different exposure levels), and adverse physical outcomes of the aging, i.e., sarcopenia, physical frailty, limited mobility, exhaustion, falls, IADL (Activities of Daily Living), ADL (Activities of Daily Living), disability, and slow gait.

The exposure was limited to different levels of coffee consumption. The outcome factors were selected to include the primary adverse physical outcomes of aging, namely frailty syndrome, sarcopenia, mobility loss, muscle loss, ADL loss, IADL loss, disability, and gait impairment, regardless of the assessment tool used (disability and functional impairment, sarcopenia, frailty phenotype, gait, and global speed, falls, physical exhaustion), or the proxy tool applied (e.g., dynamometry, bioimpedance, Short Physical Performance Battery or SPPB, sitting down and standing up test, questionnaire, and others).

The search strategy used in PubMed and MEDLINE and adapted to the other electronic sources is detailed in Supplementary Table S1. No time limit was set in the literature search, and articles were retrieved until May 2022. No language limitation was introduced. Two researchers (RZ, SM) searched the papers, reviewed titles, and abstracts of articles retrieved separately and in duplicate, checked the full text, and selected the articles for inclusion in the study. Technical reports, letters to the editor, and systematic and narrative review articles were excluded. Inter-rater reliability (IRR) was used to estimate inter-coder agreement, and then the κ statistic was used as a measure of accuracy and precision. A k coefficient of at least 0.9 was obtained in all data extraction steps based on PRISMA concepts and quality assessment steps [53].

### 4.2. Inclusion Criteria, Data Extraction, and Registration

Exposure and outcomes needed to be referred to an aging adult population (at least 40 years of age). No criterion was applied to the recruitment settings (hospital, community, or others) or health status of the study population (general population or groups with specific features). Potentially eligible articles were identified by reading the abstract and, if suitable, reading the full-text version of the articles. For each selected article, the best statistical approach was applied when considering confounding, applied to assess the magnitude of the effect of the associations. The data were cross-checked, any discrepancies were discussed, and disparities were solved by a third researcher (RS).

The following information was extracted by the two investigators (RZ, SM) separately and in duplicate in a piloted form: (1) general information about single studies (author, year of publication, country, settings, design, sample size, age); (2) level of coffee exposure (cups per day, increasing quintiles of daily consumption, or grams per day); (3) outcome(s) regarding all adverse physical outcome(s) included, regardless of the constructs or surrogate type of assessment; (4) main findings; (5) effect size of the association between exposure and outcome(s).

All references selected for retrieval from the databases were managed with the MS Excel software platform for data collection by a biostatistician (FC). Lastly, data from the selected studies stored in the database were structured as evidence tables.

### 4.3. Quality Assessment within and across Studies and Overall Quality Assessment

The methodological quality of the included studies was independently appraised by paired investigators (RZ, SM) using the National Institutes of Health Quality Assessment Toolkits for Observational Cohort and Cross-Sectional Studies [54,55]. This tool contains 14 questions that evaluate a number of factors related to the risk of bias, type I and type II errors, transparency, and confounding factors. These aspects include the study question, population, participation rate, inclusion criteria, sample size justification, time of exposure/outcome measurement, time frame, levels of exposure, defined exposure, blinded assessors, repeated exposure, defined outcomes, loss to follow-up, and confounding factors. Cross-sectional studies are not mentioned in items 6, 7, or 13. For cross-sectional and prospective investigations, the maximum possible scores were 8 and 14, respectively. Disagreements between the two investigators regarding the methodological quality of the included studies were resolved through discussion until a consensus was reached with a third investigator (RS).

## 5. Conclusions

The decline in physical functioning and disability load during aging poses a significant challenge to easing the burden of population aging on public healthcare and quality of life. In the preventive models proposed to promote a better lifestyle, there is evidence that increased coffee consumption does not implicate poor physical functioning and may indeed be protective, potentially due to bioactive load.

## Figures and Tables

**Table 1:** Selected studies investigating coffee consumption and adverse physical outcomes in aging adults (N = 10).

Authors, Year	Sample Size	Country	Age (Mean)	Study Design	Study Setting	Outcome(s)	Outcome(s) Assessment	Exposure Assessment	Major Findings
Wang T., 2020 [24]	7704	USA (America)	>60	Cross-sectional	Community US population (NHANES)	LEM	Physical Functioning Questionnaire	24-h dietary recall	Coffee consumption was inversely associated with the lower odds of functional disability in older American adults
						GPA	Physical Functioning Questionnaire	24-h dietary recall	
						LSA	Physical Functioning Questionnaire	24-h dietary recall	
						ADL disability	Physical Functioning Questionnaire	24-h dietary recall	
						IADL disability	Physical Functioning Questionnaire	24-h dietary recall	
Chung H., 2017 [25]	1781 (100% M)	Korea (Asia)	>60	Cross-sectional	Community Korean population (KNHANES)	Sarcopenia	ASMI less than two standard deviations below the gender-specific mean of this value	FFQ	Consuming at least 3 cups of coffee per day was associated with a lower prevalence of sarcopenia in elderly Korean elderly men
						Sarcopenia	ASMI less than two standard deviations below the gender-specific mean of this value	FFQ	
						Sarcopenia	ASMI less than two standard deviations below the gender-specific mean of this value	FFQ	
Kim J.H., 2017 [26]	6906 (41% M, 59% F)	Korea (Asia)	≥40	Cross-sectional	Community Korean population (KNHANES)	Sarcopenia	ASMI below the lower quintile of the study population	FFQ	Light coffee consumption was protective against sarcopenia in men
						Sarcopenia	ASMI below the lower quintile of the study population	FFQ	
						Sarcopenia	ASMI below the lower quintile of the study population	FFQ	
Kobayashi S., 2014 [27]	2121 (100% F)	Northern and western Japan (Asia)	65 + (74.7 ± 5.0)	Cross-sectional	Institutions (universities, colleges, and technical schools)	Frailty	Frailty phenotype	FFQ	Coffee intake was associated with lower odds of frailty
						Frailty	Frailty phenotype	FFQ	
						Frailty	Frailty phenotype	FFQ	
						Frailty	Frailty phenotype	FFQ	
Iwasaka C., 2021 [28]	6369 (37% M, 63% F)	Japan (Asia)	45–74 years	Cross-sectional	Community	Sarcopenia dimension	SMI (bioimpedance)	FFQ	A significant positive association was found between coffee consumption and SMI. The relationship between coffee consumption and grip strength did not reach statistical significance; however, a positive trend was observed
						Sarcopenia dimension	SMI (bioimpedance)	FFQ	
						Sarcopenia dimension	SMI (bioimpedance)	FFQ	
						Sarcopenia dimension	Grip Strength (dynamometry)	FFQ	
						Sarcopenia dimension	Grip Strength (dynamometry)	FFQ	
						Sarcopenia dimension	Grip Strength (dynamometry)	FFQ	
Huang W.C., 2021 [29]	1115	Taiwan (Asia)	65+	Cross-sectional	Community	Frailty	FRAIL scale	FFQ	Frail subjects had significantly lower daily consumption of coffee
Machado-Fragua M., 2018 [30]	2073	Spain (Europe)	60+	Longitudinal, 7-year	Community (Seniors-ENRICA cohort)	Impaired agility	Single question from the Rosow and Breslau scale: “On an average day with your current health, would you be limited in bending and kneeling?”	FFQ	Habitual coffee consumption was not associated with increased risk of functional impairment, and it might even be beneficial in women and those with hypertension, obesity or diabetes
						Impaired agility	Single question from the Rosow and Breslau scale: “On an average day with your current health, would you be limited in bending and kneeling?”	FFQ	
	2062					Impaired mobility	Responding “a lot” to any of the following questions also from the Rosow and Breslau scale: “On an average day with your current health, would you be limited in the following activities: (1) picking up or carrying a shopping bag?; (2) climbing one flight of stairs?; (3) walking several city blocks (a few 100 m)?”	FFQ	
						Impaired mobility	Responding “a lot” to any of the following questions also from the Rosow and Breslau scale: “On an average day with your current health, would you be limited in the following activities: (1) picking up or carrying a shopping bag?; (2) climbing one flight of stairs?; (3) walking several city blocks (a few 100 m)?”	FFQ	
	1653					Impaired overall physical function	≥10-point decrease from baseline to follow-up in the physical component summary score of the 12-item short-form health survey (SF-12)	FFQ	
						Impaired overall physical function	≥10-point decrease from baseline to follow-up in the physical component summary score of the 12-item short-form health survey (SF-12)	FFQ	
	2262					Impaired lower extremity function	Short Physical Performance Battery (SPPB)	FFQ	
						Impaired lower extremity function	Short Physical Performance Battery (SPPB)	FFQ	
	1714					Frailty	Frailty phenotype by Fried	FFQ	
						Frailty	Frailty phenotype by Fried	FFQ	
	1564					IADL disability	Lawton and Brody Scale	FFQ	
						IADL disability	Lawton and Brody Scale	FFQ	
	1756					ADL disability	Katz Scale	FFQ	
						ADL disability	Katz Scale	FFQ	
Verlinden V.J.A., 2016 [31]	2546 (1128 M, 1418 F)	Netherlands (Europe)	45+	Cross-sectional	Community	Global Gait	Average of seven gait domains: Rhythm, Phases, Variability, Pace, Tandem, Turning, and Base of Support	FFQ	In a community-dwelling population, consuming more than 1 cup of coffee and 1–3 glasses of alcohol relate to better gait
						Global Gait	Average of seven gait domains: Rhythm, Phases, Variability, Pace, Tandem, Turning, and Base of Support	FFQ	
						Gait speed (m/s)	5.79-m-long electronic walkway	FFQ	
						Gait speed (m/s)	5.79-m-long electronic walkway	FFQ	
Machado-Fragua M.D., 2019 [32]	2964	Spain (Europe)	60+	Cross-sectional	Community (Seniors-ENRICA cohort)	Falls	Asking participants: “How many times have you fallen down since the last interview?” and using the following outcomes in our analyses: ≥1 fall, injurious fall, and ≥1 fall with fracture	FFQ	Habitual coffee consumption was associated with lower risk of falling in older adults in Spain and the United Kingdom
						Falls	Asking participants: “How many times have you fallen down since the last interview?” and using the following outcomes in our analyses: ≥1 fall, injurious fall, and ≥1 fall with fracture	FFQ	
		UK (Europe)			Community (UK Biobank study)	Falls	Asking the participants “In the last year have you had any falls?” The possible answers were “no falls”, “only one fall”, and “more than one fall”.	FFQ	
						Falls	Asking the participants “In the last year have you had any falls?” The possible answers were “no falls”, “only one fall”, and “more than one fall”.	FFQ	
Jyvakorpi S.K., 2020 [33]	126	Finland (Europe)	60+	Cross-sectional	Hospital	Gait speed (m/s)	4-m walk, m/s	3-day food diaries	Coffee consumption was positively associated with higher gait speed, grip strength, SPPB score, and chair rise points
						Gait speed (m/s)	4-m walk, m/s	3-day food diaries	
						Gait speed (m/s)	4-m walk, m/s	3-day food diaries	
						SPPB	SPPB. Ability to stand for 10 sec with feet in 3 different positions: (1) together side-by-side, semi-tandem, and tandem; (2) two timed trials of a 3-m or 4-m walk (fastest recorded); (3) time to rise from a chair five times	3-day food diaries	
						SPPB	SPPB. Ability to stand for 10 sec with feet in 3 different positions: (1) together side-by-side, semi-tandem, and tandem; (2) two timed trials of a 3-m or 4-m walk (fastest recorded); (3) time to rise from a chair five times	3-day food diaries	
						SPPB	SPPB. Ability to stand for 10 sec with feet in 3 different positions: (1) together side-by-side, semi-tandem, and tandem; (2) two timed trials of a 3-m or 4-m walk (fastest recorded); (3) time to rise from a chair five times	3-day food diaries	
						Chair rise	Chair rise test (points): stand up repeatedly from a chair for 30 s	3-day food diaries	
						Chair rise	Chair rise test (points): stand up repeatedly from a chair for 30 s	3-day food diaries	
						Chair rise	Chair rise test (points): stand up repeatedly from a chair for 30 s	3-day food diaries	
						Sarcopenia dimension	Grip Strength (dynamometry)	3-day food diaries	

Abbreviations: LEM: lower extremity mobility; GPA: general physical activity; LSA: leisure and social activities; ADL: activities of daily living; IADL: instrumental activities of daily living; FFQ: Food Frequency Questionnaire; ASMI: Appendicular Skeletal Muscle Mass Index; SMI: Skeletal Muscle Index; SPPB: Short Physical Performance Batter.

**Table 2:** Summary of findings on the relationship between coffee consumption and adverse physical outcomes (N = 10).

Authors, Year	Sample Size (M/F)	Outcome	Level of Exposure	Strength of the Association	Major Findings
Wang T., 2020 [24]	7704	LEM	<1 cups/day	Lower extremity mobility levels across three categories of increasing coffee consumption (0 to <1, 1 to <2, and >2) versus no consumption: adj OR: 0.74 (95% CI 0.57–0.96), adj OR: 0.79 (95% CI 0.59–1.05), and adj OR: 0.67 (95% CI 0.50–0.91). *p* for trend: 0.084	Coffee consumption is inversely associated with the lower odds of functional disability in older American adults
			<2 cups/day		
			>2 cups/day		
		GPA	<1 cups/day	General physical activity levels across three categories of increasing coffee consumption (0 to <1, 1 to <2, and >2) versus no consumption: adj OR: 0.86 (95% CI 0.67–1.10), adj OR: 0.82 (95% CI 0.62–1.08), adj OR: 0.65 (95% CI 0.47–0.88). *p* for trend: 0.015	
			<2 cups/day		
			>2 cups/day		
		LSA	<1 cups/day	Leisure and social activities levels across three categories of increasing coffee consumption (0 to <1, 1 to <2, and >2) versus no consumption: adj OR: 0.93 (95% CI 0.68–1.26), adj OR: 0.96 (95% CI 0.69–1.34), adj OR: 0.61 (95% CI 0.45–0.83). *p* for trend 0.006	
			<2 cups/day		
			>2 cups/day		
		ADL disability	<1 cups/day	Activities of daily living across three categories of increasing coffee consumption (0 to <1, 1 to <2, and >2) versus no consumption: adj OR: 0.88 (95% CI 0.65–1.19), adj OR: 0.95 (95% CI 0.69–1.30), adj OR: 0.70 (95% CI 0.50–1.01). *p* for trend 0.088	
			<2 cups/day		
			>2 cups/day		
		IADL disability	<1 cups/day	Instrumental activities of daily living across three categories of increasing coffee consumption (0 to <1, 1 to <2, and >2) versus no consumption: adj OR: 0.77 (95% CI 0.57–1.03), adj OR: 0.72 (95% CI 0.54–0.95), adj OR: 0.59 (95% CI 0.44–0.78). *p* for trend 0.001	
			<2 cups/day		
			>2 cups/day		
Chung H., 2017 [25]	1781 (100% M)	Sarcopenia	1 cup/day	Logistic regression analysis between categories of increasing daily coffee consumption (1, 2, and 3 or more cups/day) versus fewer than 1 cup/day and risk of sarcopenia: adj OR: 0.69 (95% CI 0.39–1.24), adj OR: 0.60 (95% CI 0.32–1.12), adj OR: 0.44 (95% CI 0.21–0.94). *p* for trend: 0.026	Consuming at least 3 cups of coffee per day was associated with a lower prevalence of sarcopenia in elderly Korean men
			2 cups/day		
			≥3 cups/day		
Kim J.H., 2017 [26]	6906 (41% M, 59% F)	Sarcopenia	1 cup/day	Logistic regression analysis between categories of increasing daily coffee consumption (1, 2, and 3 or more cups/day) versus less than 1 cup/day and risk of sarcopenia: adj OR 0.69 (95% CI 0.50–0.94), adj OR: 1.07 (95% CI 0.79–1.45), adj OR: 0.85 (95% CI 0.60–1.22) in males, and adj OR: 0.87 (95% CI 0.69–1.10), adj OR: 0.88 (95% CI 0.68–1.15), adj OR: 0.77 (95% CI 0.56–1.06) in males	Light coffee consumption was protective against sarcopenia in men
			2 cups/day		
			≥3 cups/day		
Kobayashi S., 2014 [27]	2121 (100% F)	Physical Frailty	2nd quintile of consumption (11.3–44.6 g/day)	Linear regression analysis between quintiles of increasing daily coffee consumption (grams/day) versus the lowest quintile and risk of physical frailty: adj OR: 0.66 (95% CI 0.46–0.96) for the 2nd quintile, adj OR: 0.77 (95% CI 0.54, 1.10) for the 3rd quintile, adj OR: 0.60 (95% CI 0.41, 0.87) for the 4th quintile, and adj OR: 0.48 (95% CI 0.32, 0.72) for the highest quintile	Coffee intake was associated with lower odds of frailty
			3rd quintile of consumption (44.6–140 g/day)		
			4th quintile of consumption (140–174 g/day)		
			5th quintile of consumption (>174 g/day)		
Iwasaka C., 2021 [28]	6369 (37% M, 63% F)	Sarcopenia dimension (SMI)	<1 cup/day	Adjusted means and their 95% confidence intervals of skeletal muscle mass index according to increasing daily coffee consumption (<1, 1–2, 3 or more cups/day) compared to no consumption: adj mean 7.07 (95% CI 7.08–7.14), adj mean 7.12 (95% CI 7.09–7.14), and adj mean 7.14 (95% CI 7.11–7.17) in males, and adj mean 23.9 (95% CI 23.7–24.1), adj mean 23.8 (95% CI 23.6–24), and adj mean 23.7 (95% CI 23.4–23.9) in females	A significant positive association was found between coffee consumption and SMI. The relationship between coffee consumption and grip strength did not reach statistical significance; however, a positive trend was observed
			1–2 cups/day		
			≥3 cups/day		
		Sarcopenia dimension (HGS)	<1 cup/day	Adjusted means and their 95% confidence intervals of hand grip strength according to increasing daily coffee consumption (<1, 1–2, 3 or more cups/day) compared to no consumption: adj mean 38.1 (95% CI 37.7–38.6), adj mean 38.3 (95% CI 37.9–38.6), adj mean 38.7 (95% CI 38.2–39.1) in males, and adj mean: 23.9 (95% CI 23.7–24.1), adj mean: 23.8 (95% CI 23.6–24), adj mean: 23.7 (95% CI 23.4–23.9) in females	
			1–2 cups/day		
			≥3 cups/day		
Huang W.C., 2021 [29]	1115	Physical Frailty	Daily frequency	Significant difference in daily frequency of coffee consumption across frailty categories: 0.27 ± 0.16 (frail) vs. 0.30 ± 0.05 (pre-frail) vs. 0.34 ± 0.04 (robust) times/day. *p* < 0.05	Frail subjects had significantly lower daily consumption of coffee
Machado-Fragua M., 2018 [30]	2073	Impaired agility	1 cup/day	Hazard ratio (95% CI) of impaired agility according to increasing coffee consumption (1 and 2 or more cups/day) compared to non-coffee drinkers: HR: 0.91 (95% CI 0.77–1.09) and HR: 0.86 (95% CI 0.67–1.10). *p* for trend 0.19	Habitual coffee consumption was not associated with increased risk of functional impairment
			≥2 cups/day		
	2062	Impaired mobility	1 cup/day	Hazard ratio (95% CI) of impaired mobility according to increasing coffee consumption (1 and 2 or more cups/day) compared to non-coffee drinkers: HR: 0.82 (95% CI 0.66–1.01) and HR: 0.82 (95% CI 0.61–1.09). *p* for trend 0.07	
			≥2 cups/day		
	1653	Impaired overall physical function	1 cup/day	Hazard ratio (95% CI) of impaired overall physical function according to increasing coffee consumption (1 and 2 or more cups/day) compared to non-coffee drinkers: HR: 0.98 (95% CI 0.81–1.18) and HR: 1.03 (95% CI 0.80–1.33). *p* for trend 0.88	
			≥2 cups/day		
	2262	Impaired lower extremity function	1 cup/day	Hazard ratio (95% CI) of impaired lower extremity function according to increasing coffee consumption (1 and 2 or more cups/day) compared to non-coffee drinkers: HR: 1.21 (95% CI 0.97–1.50) and HR: 1.02 (95% CI 0.75–1.38). *p* for trend 0.45	
			≥2 cups/day		
	1714	Physical Frailty	1 cup/day	Hazard ratio (95% CI) of frailty according to increasing coffee consumption (1 and 2 or more cups/day) compared to non-coffee drinkers: HR: 1.16 (95% CI 0.85–1.60) and HR: 1.23 (95% CI 0.80–1.90). *p* for trend 0.25	
			≥2 cups/day		
	1564	IADL disability	1 cup/day	Hazard ratio (95% CI) of IADL disability according to increasing coffee consumption (1 and 2 or more cups/day) compared to non-coffee drinkers: HR: 0.79 (95% CI 0.55–1.13) and HR: 0.93 (95% CI 0.56–1.53). *p* for trend 0.46	
			≥2 cups/day		
	1756	ADL disability	1 cup/day	Hazard ratio (95% CI) of ADL disability according to increasing coffee consumption (1 and 2 or more cups/day) compared to non-coffee drinkers: HR: 0.78 (95% CI 0.62–0.99) and HR: 1.07 (95% 0.78–1.45). *p* for trend 0.66	
			≥2 cups/day		
Verlinden V.J.A., 2016 [31]	2546 (1128 M, 1418 F)	Global Gait	1–3 cups/day	Differences in standard deviation of global gait (95% CI) for increasing categories of coffee consumption (1 to 3, and 3 or more cups/day) compared to 1 or fewer cup/day: 0.13 SD (95% CI 0.01–0.25) and 0.18 SD (95% CI 0.08–0.28)	In a community-dwelling population, consuming >1 cup of coffee relate to better gait
			>3 cups/day		
		Gait speed (m/s)	1–3 cups/day	Differences in cm/s of gait speed (95% CI) for increasing categories of coffee consumption (1 to 3, and 3 or more cups/day) compared to 1 or fewer cup/day: 2.74 cm/s (95% CI 0.67–4.80) and 2.63 cm/s (95% CI 0.80–4.45)	
			>3 cups/day		
Machado-Fragua M.D., 2019 [32]	2964	Falls	1 cup/day	Hazard ratios (95% CIs) for the association between increasing coffee consumption (1 and 2 or more cups/day) and the risk of ≥1 fall compared to <1 cup/day: HR: 0.88 (95% CI 0.73–1.07) and HR: 0.79 (95% CI 0.63, 0.98). *p* for trend 0.03	Habitual coffee consumption was associated with lower risk of falling in older adults in Spain and the United Kingdom
			≥2 cups/day		
			1 cup/day	Hazard ratios (95% CIs) for the association between increasing coffee consumption (1 and 2 or more cups/day) and the risk of ≥1 fall compared to <1 cup/day: HR: 0.61 (95% CI 0.37–0.98) and HR: 0.64 (95% CI 0.39–1.03). *p* for trend 0.13	
			≥2 cups/day		
Jyvakorpi S.K. 2020 [33]	126	Gait speed (m/s)	<110 g/day	Linear association between coffee consumption and gait speed (*p* = 0.003)	Coffee consumption was positively associated with higher gait speed, handgrip strength, SPPB score, and chair rise points
			110–130 g/day		
			>330 g/day		
		SPPB	<110 g/day	Linear association between coffee consumption and SPPB-test scores (*p* = 0.035)	
			110–130 g/day		
			>330 g/day		
		Chair rise	<110 g/day	Linear association between coffee consumption and chair rise points (*p* = 0.043)	
			110–130 g/day		
			>330 g/day		
		Sarcopenia dimension (HGS)	<110 g/day	Linear, non-significant association between coffee consumption and handgrip strength (*p* = 0.856)	
			110–130 g/day		
			>330 g/day		

Abbreviations: LEM: lower extremity mobility; GPA: general physical activity; LSA: leisure and social activities; ADL: activities of daily living; IADL: instrumental activities of daily living; FFQ: Food Frequency Questionnaire; HGS: Handgrip Strength; ASMI: Appendicular Skeletal Muscle Mass Index; SMI: Skeletal Muscle Index; SPPB: Short Physical Performance Battery.

## Supplementary Materials

The following supporting information can be downloaded at: https://www.mdpi.com/article/10.3390/metabo12070654/s1. Table S1: Overview of quality ratings within selected studies. (N = 10). Table S2: Search strategy used in the US National Library of Medicine (PubMed) and Medical Literature Analysis and Retrieval System Online (MEDLINE) and adapted to the other sources, according to selected descriptors.

## References

[1] (2007). World Health Organization Global Age-Friendly Cities: A Guide.

[2] Miller E.A., Weissert W.G. (2000). Predicting Elderly People’s Risk for Nursing Home Placement, Hospitalization, Functional Impairment, and Mortality: A Synthesis. Med. Care Res. Rev..

[3] Castellana F., Lampignano L., Bortone I., Zupo R., Lozupone M., Griseta C., Daniele A., De Pergola G., Giannelli G., Sardone R. (2021). Physical Frailty, Multimorbidity, and All-Cause Mortality in an Older Population From Southern Italy: Results from the Salus in Apulia Study. J. Am. Med. Dir. Assoc..

[4] Fried L.P., Tangen C.M., Walston J., Newman A.B., Hirsch C., Gottdiener J., Seeman T., Tracy R., Kop W.J., Burke G. (2001). Frailty in Older Adults: Evidence for a Phenotype. J. Gerontol. A Biol. Sci. Med. Sci..

[5] Fried L.P., Ferrucci L., Darer J., Williamson J.D., Anderson G. (2004). Untangling the Concepts of Disability, Frailty, and Comorbidity: Implications for Improved Targeting and Care. J. Gerontol. A Biol. Sci. Med. Sci..

[6] Seidel D., Brayne C., Jagger C. (2011). Limitations in Physical Functioning among Older People as a Predictor of Subsequent Disability in Instrumental Activities of Daily Living. Age Ageing.

[7] Collard R.M., Boter H., Schoevers R.A., Oude Voshaar R.C. (2012). Prevalence of Frailty in Community-Dwelling Older Persons: A Systematic Review. J. Am. Geriatr. Soc..

[8] Seeman T.E., Merkin S.S., Crimmins E.M., Karlamangla A.S. (2010). Disability Trends among Older Americans: National Health And Nutrition Examination Surveys, 1988–1994 and 1999–2004. Am. J. Public Health.

[9] Zupo R., Castellana F., De Nucci S., Sila A., Aresta S., Buscemi C., Randazzo C., Buscemi S., Triggiani V., De Pergola G. (2022). Role of Dietary Carotenoids in Frailty Syndrome: A Systematic Review. Biomedicines.

[10] Pilleron S., Ajana S., Jutand M.-A., Helmer C., Dartigues J.-F., Samieri C., Féart C. (2017). Dietary Patterns and 12-Year Risk of Frailty: Results From the Three-City Bordeaux Study. J. Am. Med. Dir. Assoc..

[11] Zupo R., Castellana F., Guerra V., Donghia R., Bortone I., Griseta C., Lampignano L., Dibello V., Lozupone M., Coelho-Júnior H.J. (2021). Associations between Nutritional Frailty and 8-Year All-Cause Mortality in Older Adults: The Salus in Apulia Study. J. Intern. Med..

[12] Sandoval-Insausti H., Blanco-Rojo R., Graciani A., López-García E., Moreno-Franco B., Laclaustra M., Donat-Vargas C., Ordovás J.M., Rodríguez-Artalejo F., Guallar-Castillón P. (2020). Ultra-Processed Food Consumption and Incident Frailty: A Prospective Cohort Study of Older Adults. J. Gerontol. A Biol. Sci. Med. Sci..

[13] de Moraes M.B., Avgerinou C., Fukushima F.B., Vidal E.I.O. (2021). Nutritional Interventions for the Management of Frailty in Older Adults: Systematic Review and Meta-Analysis of Randomized Clinical Trials. Nutr. Rev..

[14] Gunter M.J., Murphy N., Cross A.J., Dossus L., Dartois L., Fagherazzi G., Kaaks R., Kühn T., Boeing H., Aleksandrova K. (2017). Coffee Drinking and Mortality in 10 European Countries: A Multinational Cohort Study. Ann. Intern. Med..

[15] Castellana F., De Nucci S., De Pergola G., Di Chito M., Lisco G., Triggiani V., Sardone R., Zupo R. (2021). Trends in Coffee and Tea Consumption during the COVID-19 Pandemic. Foods.

[16] Torres-Collado L., García-de la Hera M., Navarrete-Muñoz E.M., Compañ-Gabucio L.M., Gonzalez-Palacios S., Vioque J. (2018). Coffee Drinking and Associated Factors in an Elderly Population in Spain. Int. J. Environ. Res. Public Health.

[17] Nuhu A.A. (2014). Bioactive Micronutrients in Coffee: Recent Analytical Approaches for Characterization and Quantification. ISRN Nutr..

[18] Mitchell D.C., Knight C.A., Hockenberry J., Teplansky R., Hartman T.J. (2014). Beverage Caffeine Intakes in the U.S. Food Chem. Toxicol..

[19] Lopez-Garcia E., van Dam R.M., Rajpathak S., Willett W.C., Manson J.E., Hu F.B. (2006). Changes in Caffeine Intake and Long-Term Weight Change in Men and Women. Am. J. Clin. Nutr..

[20] Wang A., Wang S., Zhu C., Huang H., Wu L., Wan X., Yang X., Zhang H., Miao R., He L. (2016). Coffee and Cancer Risk: A Meta-Analysis of Prospective Observational Studies. Sci. Rep..

[21] van Dam R.M., Hu F.B. (2005). Coffee Consumption and Risk of Type 2 Diabetes: A Systematic Review. JAMA.

[22] Loopstra-Masters R.C., Liese A.D., Haffner S.M., Wagenknecht L.E., Hanley A.J. (2011). Associations between the Intake of Caffeinated and Decaffeinated Coffee and Measures of Insulin Sensitivity and Beta Cell Function. Diabetologia.

[23] Ding M., Bhupathiraju S.N., Satija A., van Dam R.M., Hu F.B. (2014). Long-Term Coffee Consumption and Risk of Cardiovascular Disease: A Systematic Review and a Dose-Response Meta-Analysis of Prospective Cohort Studies. Circulation.

[24] Wang T., Wu Y., Wang W., Zhang D. (2021). Association between Coffee Consumption and Functional Disability in Older US Adults. Br. J. Nutr..

[25] Chung H., Moon J.H., Kim J.I., Kong M.H., Huh J.S., Kim H.J. (2017). Association of Coffee Consumption with Sarcopenia in Korean Elderly Men: Analysis Using the Korea National Health and Nutrition Examination Survey, 2008–2011. Korean J. Fam. Med..

[26] Kim J.-H., Park Y.S. (2017). Light Coffee Consumption Is Protective against Sarcopenia, but Frequent Coffee Consumption Is Associated with Obesity in Korean Adults. Nutr. Res..

[27] Kobayashi S., Asakura K., Suga H., Sasaki S. (2014). Three-generation Study of Women on Diets and Health Study Groups Inverse Association between Dietary Habits with High Total Antioxidant Capacity and Prevalence of Frailty among Elderly Japanese Women: A Multicenter Cross-Sectional Study. J. Nutr. Health Aging.

[28] Iwasaka C., Yamada Y., Nishida Y., Hara M., Yasukata J., Miyoshi N., Shimanoe C., Nanri H., Furukawa T., Koga K. (2021). Association between Habitual Coffee Consumption and Skeletal Muscle Mass in Middle-Aged and Older Japanese People. Geriatr. Gerontol. Int..

[29] Huang W.-C., Huang Y.-C., Lee M.-S., Chang H.-Y., Doong J.-Y. (2021). Frailty Severity and Cognitive Impairment Associated with Dietary Diversity in Older Adults in Taiwan. Nutrients.

[30] Machado-Fragua M.D., Struijk E.A., Graciani A., Guallar-Castillon P., Rodríguez-Artalejo F., Lopez-Garcia E. (2019). Coffee Consumption and Risk of Physical Function Impairment, Frailty and Disability in Older Adults. Eur. J. Nutr..

[31] Verlinden V.J.A., Maksimovic A., Mirza S.S., Ikram M.A., Kiefte-de Jong J.C., Hofman A., Franco O.H., Tiemeier H., van der Geest J.N. (2016). The Associations of Alcohol, Coffee and Tobacco Consumption with Gait in a Community-Dwelling Population. Eur. J. Clin. Nutr..

[32] Machado-Fragua M.D., Struijk E.A., Ballesteros J.-M., Ortolá R., Rodriguez-Artalejo F., Lopez-Garcia E. (2019). Habitual Coffee Consumption and Risk of Falls in 2 European Cohorts of Older Adults. Am. J. Clin. Nutr..

[33] Jyväkorpi S.K., Urtamo A., Kivimäki M., Strandberg T.E. (2021). Associations of Coffee Drinking with Physical Performance in the Oldest-Old Community-Dwelling Men The Helsinki Businessmen Study (HBS). Aging Clin. Exp. Res..

[34] Sardone R., Castellana F., Bortone I., Lampignano L., Zupo R., Lozupone M., Griseta C., Dibello V., Seripa D., Guerra V. (2021). Association Between Central and Peripheral Age-Related Hearing Loss and Different Frailty Phenotypes in an Older Population in Southern Italy. JAMA Otolaryngol. Head Neck Surg..

[35] Zupo R., Castellana F., Bortone I., Griseta C., Sardone R., Lampignano L., Lozupone M., Solfrizzi V., Castellana M., Giannelli G. (2020). Nutritional Domains in Frailty Tools: Working towards an Operational Definition of Nutritional Frailty. Ageing Res. Rev..

[36] Zupo R., Castellana F., De Nucci S., Dibello V., Lozupone M., Giannelli G., De Pergola G., Panza F., Sardone R., Boeing H. (2021). Beverages Consumption and Oral Health in the Aging Population: A Systematic Review. Front. Nutr..

[37] Tatoli R., Lampignano L., Donghia R., Castellana F., Zupo R., Bortone I., De Nucci S., Campanile G., Lofù D., Vimercati L. (2022). Dietary Customs and Social Deprivation in an Aging Population From Southern Italy: A Machine Learning Approach. Front. Nutr..

[38] Guo Y., Niu K., Okazaki T., Wu H., Yoshikawa T., Ohrui T., Furukawa K., Ichinose M., Yanai K., Arai H. (2014). Coffee Treatment Prevents the Progression of Sarcopenia in Aged Mice in Vivo and in Vitro. Exp. Gerontol..

[39] Jang Y.J., Son H.J., Kim J.-S., Jung C.H., Ahn J., Hur J., Ha T.Y. (2018). Coffee Consumption Promotes Skeletal Muscle Hypertrophy and Myoblast Differentiation. Food Funct..

[40] Dirks-Naylor A.J. (2015). The Benefits of Coffee on Skeletal Muscle. Life Sci..

[41] Ludwig I.A., Clifford M.N., Lean M.E.J., Ashihara H., Crozier A. (2014). Coffee: Biochemistry and Potential Impact on Health. Food Funct..

[42] Solfrizzi V., Panza F., Imbimbo B.P., D’Introno A., Galluzzo L., Gandin C., Misciagna G., Guerra V., Osella A., Baldereschi M. (2015). Coffee Consumption Habits and the Risk of Mild Cognitive Impairment: The Italian Longitudinal Study on Aging. J. Alzheimers Dis..

[43] Gross G., Jaccaud E., Huggett A.C. (1997). Analysis of the Content of the Diterpenes Cafestol and Kahweol in Coffee Brews. Food Chem. Toxicol..

[44] Ferré S., Orrú M., Guitart X. (2013). Paraxanthine: Connecting Caffeine to Nitric Oxide Neurotransmission. J. Caffeine Res..

[45] Jäger R., Purpura M., Wells S.D., Liao K., Godavarthi A. (2022). Paraxanthine Supplementation Increases Muscle Mass, Strength, and Endurance in Mice. Nutrients.

[46] Stavric B. (1988). Methylxanthines: Toxicity to Humans. 3. Theobromine, Paraxanthine and the Combined Effects of Methylxanthines. Food Chem. Toxicol..

[47] Aubier M. (1986). Effect of Theophylline on Diaphragmatic and Other Skeletal Muscle Function. J. Allergy Clin. Immunol..

[48] Ohnishi A., Kato M., Kojima J., Ushiama H., Yoneko M., Kawai H. (2003). Differential Pharmacokinetics of Theophylline in Elderly Patients. Drugs Aging.

[49] Evans W.V. (1984). Plasma Theophylline Concentrations, Six Minute Walking Distances, and Breathlessness in Patients with Chronic Airflow Obstruction. Br. Med. J..

[50] Eaton M.L., MacDonald F.M., Church T.R., Niewoehner D.E. (1982). Effects of Theophylline on Breathlessness and Exercise Tolerance in Patients with Chronic Airflow Obstruction. Chest.

[51] Page M.J., Moher D., Bossuyt P.M., Boutron I., Hoffmann T.C., Mulrow C.D., Shamseer L., Tetzlaff J.M., Akl E.A., Brennan S.E. (2021). PRISMA 2020 Explanation and Elaboration: Updated Guidance and Exemplars for Reporting Systematic Reviews. BMJ.

[52] Morgan R.L., Whaley P., Thayer K.A., Schünemann H.J. (2018). Identifying the PECO: A Framework for Formulating Good Questions to Explore the Association of Environmental and Other Exposures with Health Outcomes. Environ. Int..

[53] Belur J., Tompson L., Thornton A., Simon M. (2021). Interrater Reliability in Systematic Review Methodology: Exploring Variation in Coder Decision-Making. Sociol. Methods Res..

[54] Koren-Hakim T., Gumieiro D.N., Drevet S. Others Quality of the Selected Observational Study Was Assessed Using the National Institutes of Health (NIH) Quality Assessment Tool for Observational Cohort and Cross-Sectional Studies. Criteria1. Was the Research Question or Objective in This Paper Clearly Stated? Criteria 2. Was the Study Population Clearly Specified and Defined? Criteria 3. Was the Participation Rate of Eligible Persons at Least 50%? Criteria 4. Were All the Subjects Selected or Recruited from the Same or Similar Populations (including the Same Time Period)? Were Inclusion and Exclusion. https://www.nhlbi.nih.gov/health-topics/study-quality-assessment-tools.

[55] Schwingshackl L., Rüschemeyer G., Meerpohl J.J. (2021). [How to interpret the certainty of evidence based on GRADE (Grading of Recommendations, Assessment, Development and Evaluation)]. Urologe A.

