# Supplementary material for: Coffee Drinking and Adverse Physical Outcomes in the Aging Adult Population: A Systematic Review
Source: Metabolites. 2022 Jul 15;12(7):654. doi: 10.3390/metabo12070654 (PMC9318773; doi:10.3390/metabo12070654)
Supplement: Supplementary file 1 [file metabolites-12-00654-s001.zip › metabolites-1802501-supplementary.pdf]

Supplementary Material

Supplementary Table S1. Overview of quality ratings within selected studies. (N=10)

Authors	**% of YES**	**% of NO**	**% of Not reported**	**% of Not Ap-plicable**	**Qual-ity***
Wang T. 2020	50	0	14	36	Poor
Chung H. 2017	50	0	14	36	Poor
Kim JH. 2017	50	0	14	36	Poor
Kobayashi S. 2014	50	0	14	36	Poor
Iwasaka C. 2021	50	0	14	36	Poor
Huang WC. 2021	36	14	14	36	Poor
Machado-Fragua M. 2018	79	0	14	7	Good
Verlinden VJA. 2016	57	0	7	36	Fair
Machado-Fragua MD. 2019	57	0	7	36	Fair
Jyvakorpi SK. 2020	43	7	14	36	Poor

*The quality was set according to the total score: 0-5 (Poor) , 6-10 (fair) and 11-14 (Good).

Supplementary Table S2. Search strategy used in the US National Library of Medicine (PubMed) and Medical Literature Analysis and Retrieval System Online (MEDLINE) and adapted to the other sources, according to selected descriptors.

Research Question: What is the association between coffee drinking and adverse physical outcomes in the adult aging population?

Sources: PubMed, MEDLINE, EMBASE, Scopus, Ovid, and Google Scholar.

Limitations: "Human"

Grey literature: https://arxiv.org/. Furthermore, https://www.base-search.net/ was used to avoid publication bias in terms of contradictory and negative results reports, especially in a grey research question such as the one we selected.

Search Date: Inception to May 31st, 2022.

N.	Searches	Results
#1	("Intake"[tiab]) OR ("Consumption"[tiab])	586,931
#2	("Sarcopenia"[tiab]) OR ("Frailty"[tiab]) OR ("Fall*"[tiab]) OR ("Gait"[tiab]) OR ("Mobility"[tiab]) OR ("ADL"[tiab]) OR ("IADL"[tiab]) OR ("Disability"[tiab]) OR ("Functional Impairment"[tiab])	666,601
#3	("Coffee"[tiab])	16,970
#4	("Review"[tiab]) OR ("Systematic review"[tiab])OR ("Narrative review"[tiab])) OR ("Meta-analysis"[tiab])	3,876,458
#5	#1 AND #2 AND #3 NOT #4	284
